# Investigation of Changes in Fatigue Damage Caused by Mean Load under Block Loading Conditions

**DOI:** 10.3390/ma14112738

**Published:** 2021-05-22

**Authors:** Roland Pawliczek, Tadeusz Lagoda

**Affiliations:** Department of Mechanics and Machine Design, Opole University of Technology, 45-271 Opole, Poland; t.lagoda@po.edu.pl

**Keywords:** block loads, cyclic stress–strain curve, mean load, fatigue damage accumulation

## Abstract

The literature in the area of material fatigue indicates that the fatigue properties may change with the number of cycles. Researchers recommend taking this into account in fatigue life calculation algorithms. The results of simulation research presented in this paper relate to an algorithm for estimating the fatigue life of specimens subjected to block loading with a nonzero mean value. The problem of block loads using a novel calculation model is presented in this paper. The model takes into account the change in stress–strain curve parameters caused by mean strain. Simulation tests were performed for generated triangular waveforms of strains, where load blocks with changed mean strain values were applied. During the analysis, the degree of fatigue damage was compared. The results of calculations obtained for standard values of stress–strain parameters (for symmetric loads) and those determined, taking into account changes in the curve parameters, are compared and presented in this paper. It is shown that by neglecting the effect of the mean strain value on the *K*′ and *n*′ parameters and by considering only the parameters of the cyclic deformation curve for *ε_m_* = 0 (symmetric loads), the ratio of the total degree of fatigue damage varies from 10% for *ε_a_* = 0.2% to 3.5% for *ε_a_* = 0.6%. The largest differences in the calculation for ratios of the partial degrees of fatigue damage were observed in relation to the reference case for the sequence of block *n*_3_, where *ε_m_* = 0.4%. The simulation results show that higher mean strains change the properties of the material, and in such cases, it is necessary to take into account the influence of the mean value on the material response under block loads.

## 1. Introduction

Material fatigue properties are described by means of standard characteristics (e.g., S–N curve or ε–N curve and cyclic stress–strain curve). They are usually obtained under laboratory testing conditions for constant-amplitude loads. Other factors affecting fatigue life, such as mean load value, notch performance, complex load conditions, and others, also require separate testing and mathematical modeling. In engineering practice, it is assumed that such factors are treated as material properties with fixed values. Studies proving the variability of these factors with the number of fatigue cycles are also available in the literature [1,2]. This effect is characteristic for cyclically unstable materials, and the literature indicates that the calculation algorithms, e.g., for fatigue life estimation, should take into account the phenomena related to the strengthening or weakening of such materials [3,4,5,6].

Block loads are a special case of operational loads. During the operation of a structure or machine, it is possible to clearly distinguish fragments with significantly different levels of repeated cyclic load. As an example, we can indicate the diametrically different loads on the wings of aircraft during flight, where several phases (stop–takeoff–cruise–landing) can be distinguished or, as another example, the diametrically different loads on large trucks when transporting minerals and aggregates. There are clearly phases of variable loads with different mean loads.

**Figure 1:**
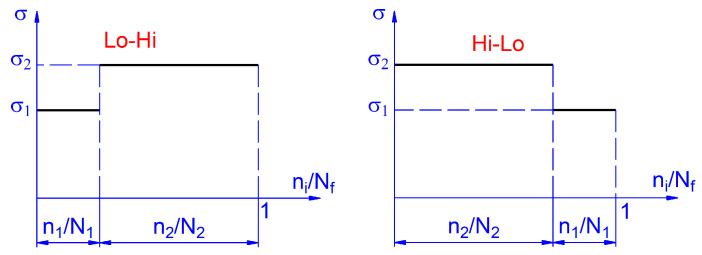
The scheme of block loads.

It becomes important to determine the impact of individual sequences on fatigue processes occurring in the material in terms of both load levels and their order. For example, the influence of Lo/Hi and Hi/Lo load sequences on material fatigue life is well known. This effect is most often studied using block loads, where the life cycle of the tested components is most often divided into two parts with significantly different levels of stress *σ* (Figure 1). The expression *n_i_*/*N_f_* represents the proportion of cycles *n_i_* with respect to the life of the *N_f_* sample. The index *i* (here, *i* = 1, 2) denotes the step in the load block.

Usually, two-state tests are carried out (Figure 1), where, for a certain number of cycles *n*_1_, the material is loaded with stress *σ*_1_ and, then, the second phase of the number of cycles *n*_2_ with stress *σ*_2_ is applied. Such tests are carried out in two ways:

1. After the first phase of loading, the second phase of testing is carried out to obtain the criterion of sample failure.
2. The sequence of cycles *n*_1_ and *n*_2_ is determined, and this load sequence is repeated until the criterion of sample failure is not reached.

The main purpose of the research is to determine the cumulative damages in the material in individual loading phases. The results of the durability tests are related to the standard fatigue characteristics of the materials obtained under constant load conditions.

**Figure 2:**
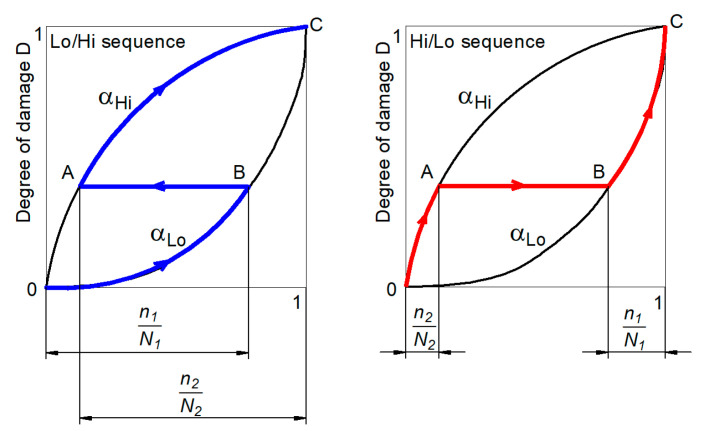
Nonlinear model of fatigue failure accumulation for Lo/Hi sequence and Hi/Lo sequence.

The use of nonlinear fatigue damage accumulation models is well described in the literature [7,8], and these results are very often the basis for more detailed analyses in the case of block loads, in which individual sequences are formed by symmetrical load cycles [9]. It is commonly recognized that the appearance of a high level of load in the first cycle increases the fatigue life as compared to the case where the stress increase occurs in the second load block. The different behaviors of the material are explained by the nonlinearity of the fatigue failure accumulation curves, where the change in the stress level is related to the change in the failure accumulation curve, as shown in Figure 2.

As shown in Figure 2, we can determine the following Equation (1):

(1) $$D_{Lo-Hi}=\frac{n_{1}}{N_{1}}+\frac{n_{2}}{N_{2}}>1\;D_{Hi-L_{0}}=\frac{n_{2}}{N_{2}}+\frac{n_{1}}{N_{1}}<1$$

Despite the fact that the load parameters in the Hi/Lo and Lo/Hi paths are the same, the sequence of load affects the value of accumulated damage. As a result, we obtain different fatigue lives. Without taking into account block loads in the design, we can affect the safety of the structure if the load path method causes greater damage than that determined from the models ignoring the influence of the load history.

Equation (2) presents a basic relationship for a nonlinear model of fatigue damage accumulation.

(2) $$D=\sum D_{i}=\sum {\left(\frac{n_{i}}{N_{i}}\right)}^{a}$$

where *α* is the exponent depending on the load level, *n_i_* is the number of cycles in a load block with stress *σ_i_*, and *N_i_* is the number of cycles to failure determined from the base fatigue characteristics under constant-amplitude loads.

It is worth pointing out that, for such loads, instability of the parameters of the cyclic strain curve was also observed [10].

Additionally, the basic research problems under fatigue conditions of block loads refer to symmetrical loads, where the mean value is zero. This allows, above all, for assessments of the impact of the load sequence on the course of fatigue phenomena and to verify the calculation models of fatigue life estimation. An interesting extension of research in block conditions was presented in [11,12], whereby load blocks create a complex state of alternating bending and torsion. The authors proposed the nonlinear damage accumulation aggregation hypothesis based on material memory.

There are few studies in the literature on block loads considering asymmetric cycles (mean load). In the case of block loads involving different mean values in each load sequence, different trends underlying the changes in fatigue life are observed. In a study by Memon et al. [13], the results of fatigue tests under two-stage block loads in Lo/Hi and Hi/Lo sequences were presented for different sequences of amplitude and mean load. The authors investigated the possibility of applying the Palmgreen–Miner hypothesis of accumulation of fatigue damage for such loads. The results of this research indicate that, while the differences in load amplitudes for subsequent blocks are large, a significant impact on the level of accumulated fatigue damage for Hi/Lo and Lo/Hi sequences can be observed and, thus, significant differences in fatigue life are obtained. Gołoś and Dębski [14] took into account the mean strain value using the total strain energy density parameter. It was observed that the mean strain values affect the energy fatigue characteristics of the material. The authors proposed a fatigue damage accumulation model using these modified characteristics. Test results on St5 steel samples showed that the additional mean strain reduces the fatigue life under block load conditions, and the authors proposed a fatigue damage accumulation model, which successfully described the test results. Some studies available in the literature [15,16,17,18] presented a wide range of fatigue tests on S355J0 steel under three-stage bending block loads. It is indicated that the effect of mean load depends on the load level and is more important for higher load amplitude values. When plastic deformation in the material is limited, the mean load value has little effect. It has also been observed that fatigue life in the case of block loads is greater compared to tests under constant-amplitude conditions.

The purpose of this work was to perform simulation tests and to assess the impact of changes in the parameters of the cyclic strain curve caused by mean strain on the degree of fatigue damage in relation to the strain amplitude.

## 2. Stress–Strain Analysis Model

For the analysis of the state of stress and strain, an algorithmic method is proposed, whereas a function of stress time histories, the calculation of the degree of fatigue damage, is based on standard fatigue characteristics.

**Figure 3:**
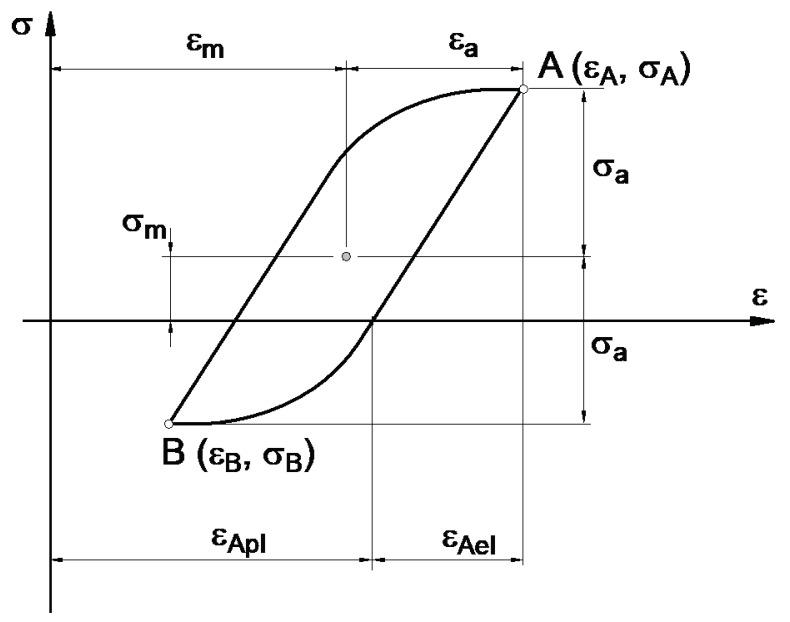
Hysteresis loop model with mean load.

The stress–strain hysteresis loop model from the literature [19], described in Figure 3, was adopted. It was assumed that the shape of the stable hysteresis loop remains unchanged under the same strain amplitude instead of presenting different mean strain values. Additionally, the stable hysteresis loop shifts in accordance with the value of the mean strain. This results in symmetry of the hysteresis loop according to the point described by mean strain *ε_m_* and mean stress *σ_m_* (see Figure 3).

**Figure 4:**
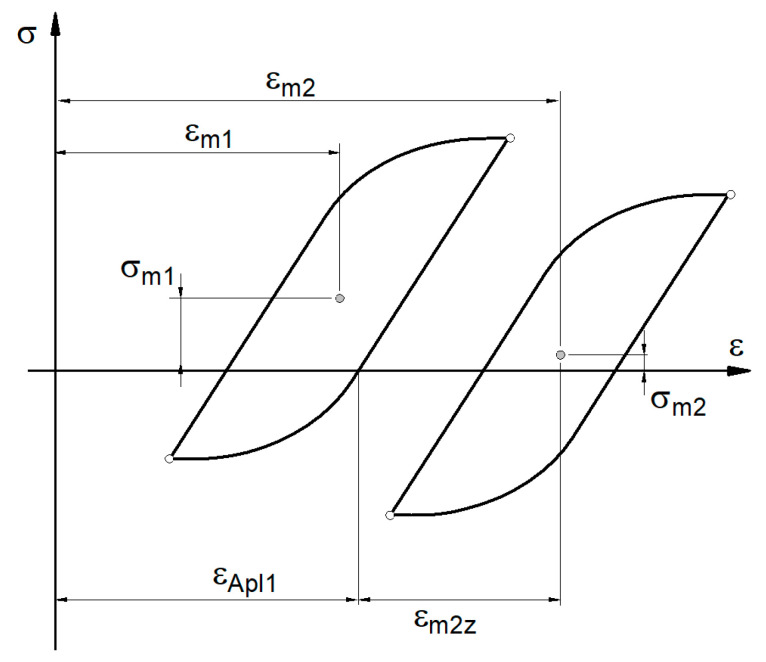
Interpretation of mean strain due to new load sequence.

It was assumed that, after changing the load conditions in the block, the current state of the material is the starting point for further operation in the subsequent load sequence. This then causes additional material deformations as a result of the increase in mean value. A graph of hysteresis loop positions is presented in Figure 4 [17].

In the general case, when the actual loop changes position, *ε_m_*_2_ represents the deformation related to the state of the material previously achieved. However, to take into account the previous load history, it was assumed that the starting point for the deformation analysis is the level of permanent deformation after the previous load blocks. Hence, for further analysis, the strain was marked as *ε_m_*_2z_.

By taking into account the assumptions presented above (Figure 3 and Figure 4), an algorithm to compute the stress history was defined, where all steps must be repeated for each sequence in the block load for the entire recorded history of strains. The algorithm is presented in Table 1. The values of stress amplitude and mean value are taken as the input data to determine fatigue failure and to estimate fatigue life with the use of standard fatigue characteristics.

## 3. Analytical Simulation

Material data were taken from the literature. Chiou and Yip [19] presented the effect of mean strain level on the cyclic stress–strain behavior of AISI 316 stainless steel. Typical applications of 316 stainless steel include the design of exhaust manifolds, heat exchangers, valve and pump parts, chemical processing equipment, and parts exposed to the marine environment. Table 2 presents the parameters of cyclic strain curves obtained for different levels of mean strain.

**Figure 5:**
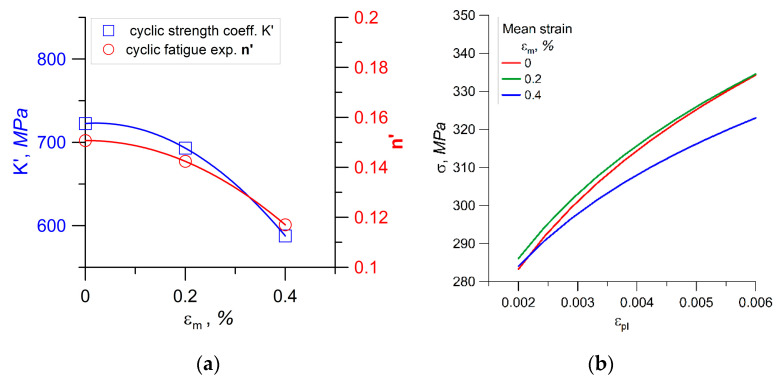
Mean strain effect on cyclic stress–strain curves: (**a**) Changes in the coefficients *K*′ and *n*′; (**b**) plastic region of stress–strain curves.

Figure 5a graphically depicts the results in Table 2. It is shown that, for a higher value of the mean strain, the effect is stronger. Stress–strain curves according to the data in Table 2 are presented in Figure 5b. For mean strains equal to 0% and 0.2%, there are no significant differences in the values of stress–strain curve parameters.

**Figure 6:**
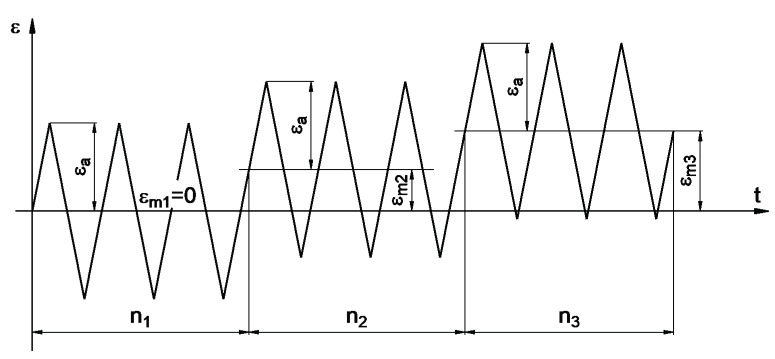
Time sequence of the simulated strains.

In the simulation tests, the degree of accumulated fatigue damage was calculated for block loads consisting of three sequences with different values of mean strain. As a baseline, a sawtooth strain waveform was used, as shown in Figure 6, where the strain amplitude has a fixed value.

The mean strain values were adopted according to Table 2, i.e., *ε_m_*_1_ = 0, *ε_m_*_2_ = 0.2%, and *ε_m_*_3_ = 0.4%. Each load block consisted of *n*_1_ = *n*_2_ = *n*_3_ = 1000 cycles. The strain amplitude was made to vary in the range *ε_a_* = 0.2–0.60%. The fatigue life N was estimated by using the approximate relationship *log(N)* = −12.88·*log(σ_a_)* − 36.38 on the basis of the data presented by Lei et al. [20].

For each level of the strain amplitude, the amplitude and mean value of the stress were calculated. Then, the fractional damage accumulation degree *D_i_* = *n_i_*/*N_i_* was computed (*i* = 1, 2, and 3 for each block load sequence), where *N_i_* is the expected fatigue life for the obtained stress state, assuming a constant-amplitude load. Then, the total degree of the damage accumulation was calculated as a sum of the partial degrees.

The calculations were made according to the algorithm in Table 1 for two cases:

- **Case A**, where the change in *K*′ and *n*′ parameters was taken into account for different values of the mean strain *e_m_*,
- **Case B**, where the influence of mean strain *e_m_* on the values of the coefficients K ‘and *n*′ was omitted, assuming their values to be *e_m_* = 0.

In Table 3, the results of calculations obtained for **Case A** and **Case B** are listed. In the tables, indices 1, 2, and 3 refer to *n*_1_, *n*_2_, and *n*_3_ in Figure 6, while letters A and B refer to Cases A and B.

In the second simulation, the influence of mean strain on the change in *n*′ and *K*′ values was omitted in the calculations. Table 4 presents the results of these simulations.

**Figure 7:**
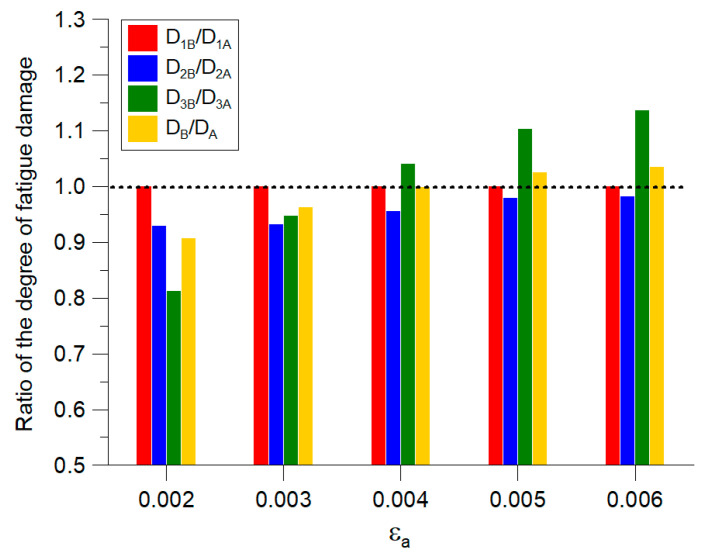
The ratio D_iB_/D_iA_ of the partial degrees of fatigue damage and the ratio D_B_/D_A_ of the total degree of fatigue damage.

In Figure 7, a comparison of the calculated degrees of damage accumulation for both cases is shown. In this figure, the ratio D_iB_/D_iA_ of the partial degrees of fatigue damage and the ratio D_B_/D_A_ of the total degree of fatigue damage for both cases are shown.

The dotted line, at level 1.0, denotes that the degrees of fatigue damage are the same for both cases and that the mean strain value has no effect. The chart indicates that, upon increasing the mean strain value, the calculation inaccuracy also increases, up to ±20% for the third part of the load sequence, where *ε_m_* = 0.4%. Meanwhile, the ratio D_B_/D_A_ of the total degree of fatigue damage varied from −10% to 3.5%. The biggest inaccuracies in fatigue life estimations were expected for stress states approaching the fatigue limit. Thus, under these conditions, the influence of mean strain value cannot be neglected.

**Figure 8:**
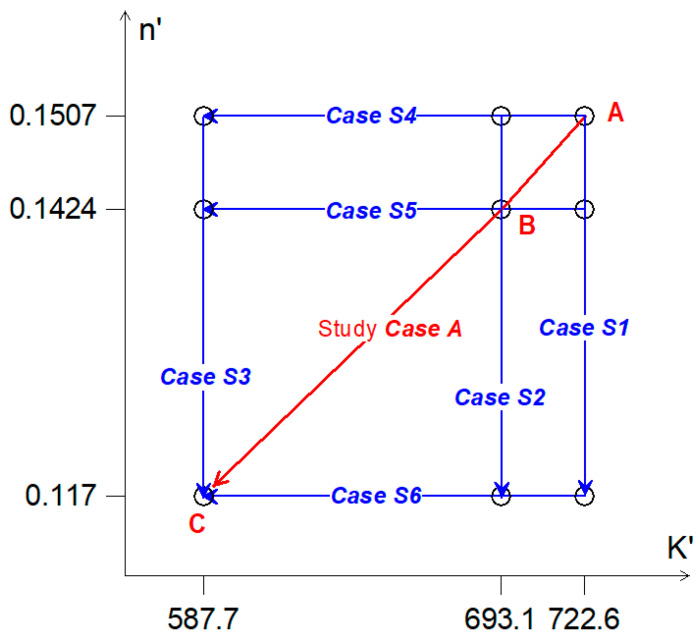
Graph of parameters for simulation cases.

Not much data exist for the problem analyzed above. Thus, it was necessary to perform an experiment to obtain Ramberg–Osgood curve parameters for different mean strains or stresses, thereby allowing the identification and evaluation of possible alternative combinations of these parameters. Taking into account the data for the analyzed material, an array of the coefficients is presented in Figure 8. It was also assumed that the cases in which the parameters *K*′ and *n*′ change according to the paths specified as Case S1 to Case S6 in Figure 8 would be analyzed. The line ABC in Figure 8, marked Study Case A, presents the previously analyzed Case A. For further simulations, this case was used as a reference.

**Figure 9:**
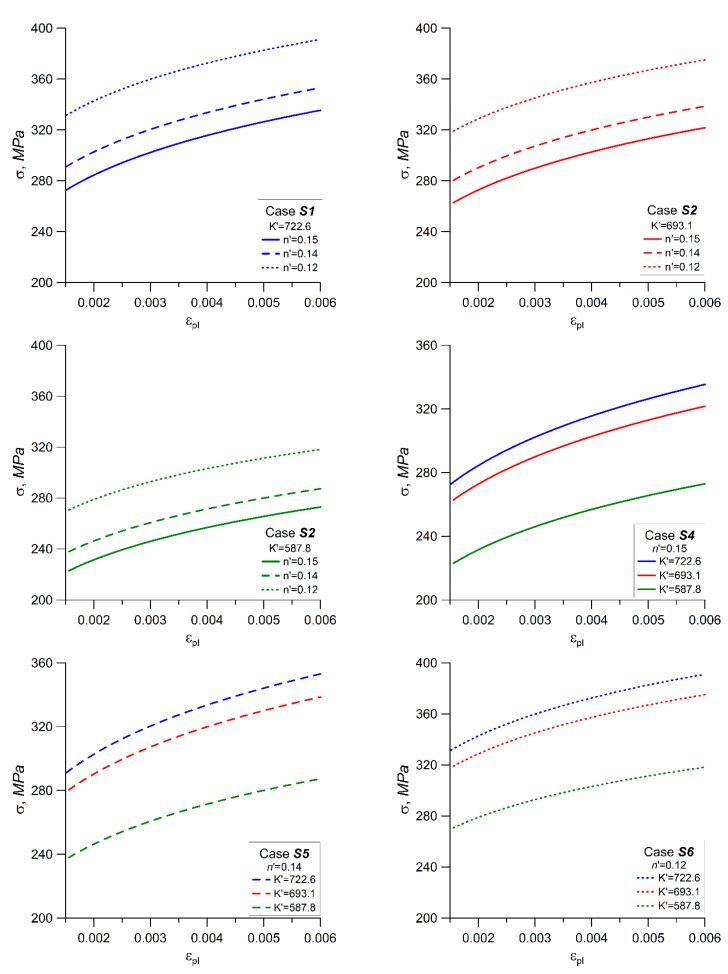
Simulated Ramberg–Osgood graphs.

**Figure 10:**
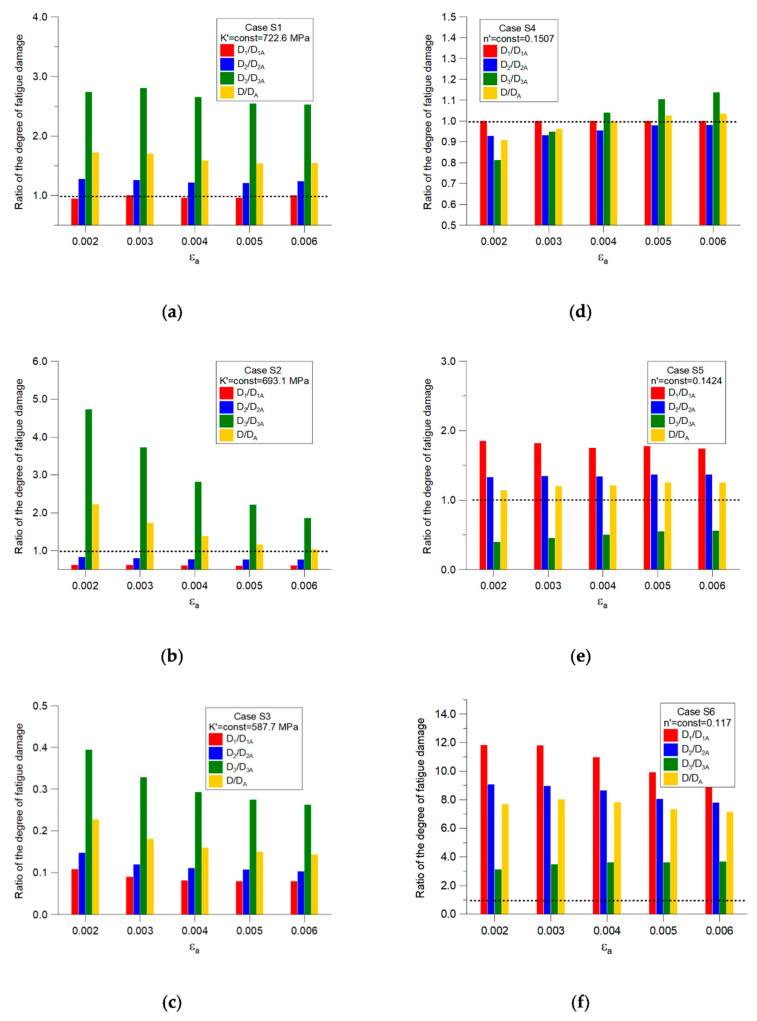
Simulation results for Cases S1–S6. (**a**) Results for Case S1, *K*′ = 722.6 MPa; (**b**) Results for Case S2, *K*′ = 693.1 MPa; (**c**) Results for Case S3, *K*′ = 587.7 MPa; (**d**) Results for Case S4, *n*′ = 0.1507; (**e**) Results for Case S5, *n*′ = 0.1424; (**f**) Results for Case S6, *n*′ = 0.1170.

The simulated Ramberg–Osgood graphs for Cases S1–S6 defined above are shown in Figure 9. The results of the simulations are presented in Figure 10. The graphs in Figure 10a–c shows cases where the cyclic strength coefficient *K*′ is independent of the mean strain, and the cyclic fatigue exponent *n*′ changes for each block sequence *n*_1_, *n*_2_, and *n*_3_ (see Figure 6) depending on the mean strain. By increasing the value of the *K*′ coefficient, it can be noted that the largest changes in relation to the reference case (Case A, Table 3) were observed for the third load sequence *n*_3_, where the mean strain value was the largest (*ε_m_* = 0.4%). For *K*′ = 722.6 MPa, the ratio of the total degree of fatigue damage was D/D_A_ = 1.5–17, which gives a good approximation to the reference case. In the case of *K*′ = 587.7 MPa, the accumulated fatigue damage was four times smaller than the reference value.

The graphs in Figure 10d–f show the opposite situation. Here, it was assumed that the coefficient *n*′ is independent of the mean strain, while *K*′ decreases with increasing mean strain values in sequences *n*_1_, *n*_2_, and *n*_3_.

It can be seen that the best results of calculations in relation to the reference Case A were obtained when *n*′ = 0.1507 for ratios of both the total degree of fatigue damage (D/D_A_ = 0.9–1.04) and the partial degrees of fatigue damage. Decreasing the value of *n*′ for cases S5 and S6 caused a decrease in the effect on the accumulation of fatigue damage for the *n*_3_ block sequence (*ε_m_* = 0.4%). For the S5 sequence, the total damage ratio was determined to be D/D_A_ = 1.1–1.3, whereas, for Case S6, this ratio was D/D_A_ = 7.5–8.0.

## 4. Summary and Conclusions

This paper presented the results of simulation tests of block loads with a nonzero mean strain value. The calculations were made using an algorithmic method, which took into account the changes in the cyclic strain curve parameters depending on the mean strain value. The calculations were made on the basis of research available in the literature on AISI 316 steel. The material response for various combinations of cyclic strength coefficient *K*′ and cyclic fatigue exponent *n*′ was analyzed. According to the results, the following conclusions can be drawn:

- When neglecting the effect of the mean strain value on the *K*′ and *n*′ parameters and considering only the parameters of the cyclic deformation curve for *ε_m_* = 0 (symmetric loads), the ratio of the total degree of fatigue damage varied from 10% for *ε_a_* = 0.2% to 3.5% for *ε_a_* = 0.6%. The largest differences in the calculation of the ratio of the partial degrees of fatigue damage in relation to the reference case were observed for sequence block *n*_3_, where *ε_m_* = 0.4%.
- When assuming the independence of parameter *K*′ from the mean strain value, the worst calculation results in relation to the reference Case A were obtained for *K*′ = 587.7 MPa, where the total degree of fatigue damage was, on average, four times lower than the reference case. For these simulations, the largest calculation inaccuracy was also related to the *n*_3_ block load sequence, where the mean strain value was the largest (0.4%).
- When considering the independence of parameter *n*′ from the mean strain value, the best results in terms of the degree of fatigue damage calculation were achieved for *n*′ = 0.1507 (obtained for a symmetric load, *ε_m_* = 0). The differences in the ratios of partial and total degrees of fatigue damage compared to the reference case were in the range of −20% to 4%. Similar results were obtained for Case B, where parameters *K*′ and *n*′ characterized the cyclic strain curve for symmetric loads.
- It can be concluded that the third sequence *n*_3_, where the biggest mean strain value was applied (*ε_m_* = 0.4%), led to the largest inaccuracy. A higher value of mean strains, thus, increases the sensitivity of the algorithm toward applied parameters *K*′ and *n*′.

## Figures and Tables

**Table 1:** Algorithm to calculate the stress history.

Step No.	Description of Operation	Equation
1.	Material properties: *E*, Young’s modulus; *K*′, cyclic strength coefficient; *n*′, cyclic strain-hardening exponent.	
2.	Input data: *ε_A_*, *ε_B_*, *ε_a_*, and *ε_m_*	
3.	alculation of *σ_A_* for given *K*′ and *n*′ (*)	$\varepsilon_{a}=\frac{\sigma_{A}}{E}+{\left(\frac{\sigma_{A}}{K^{\prime }}\right)}^{\frac{1}{n^{\prime }}}$
	(*) Values of the coefficients *K*′ and *n*′ depend on the current values of the average strain *ε_m_*. However, this requires additional tests to determine the functions *K*′ = *f* (*ε_m_*) and *n*′ = *f* (*ε_m_*).	
4.	alculation of *ε_Apl_*	$\varepsilon_{Apl}=\varepsilon_{A}-\frac{\sigma_{A}}{E}$
5.	alculation of *σ_a_* for given *K* and *n* obtained for *ε_m_* = 0	$\varepsilon_{a}=\frac{\sigma_{a}}{E}+{\left(\frac{\sigma_{a}}{K}\right)}^{\frac{1}{n}}$
6.	alculation of *ε_apl_*	$\varepsilon_{apl}=\varepsilon_{a}-\frac{\sigma_{a}}{E}$
7.	Calculation of mean stress *σ_m_*	$\sigma_{m}=E\left(\varepsilon_{miz}+\varepsilon_{ap\;}-\varepsilon_{Apl}\right)$ *i* refers to the next load sequence
8.	Resulting parameters (*σ_a_*, *σ_m_*)	

**Table 2:** *K*′ and *n*′ factors for different mean strain levels.

*ε_m_* (%)	0	0.2	0.4
*K*′ (MPa)	722.6	693.1	587.7
*n*′	0.1507	0.1424	0.117

**Table 3:** Case A: Result of simulation considering the effect of mean strain.

ε_a_	σ_a1_ (MPa)	σ_a2_ (MPa)	σ_m2_ (MPa)	σ_a3_ (MPa)	σ_m3_ (MPa)	D_1A_	D_2A_	D_3A_	D_A_
0.0020	246	249	3	253	7	0.0026	0.0028	0.0032	0.0086
0.0030	275	278	3	277	2	0.0109	0.0117	0.0115	0.0341
0.0040	294	296	2	292	−2	0.0258	0.0270	0.0248	0.0776
0.0050	308	309	1	303	−5	0.0470	0.0480	0.0426	0.1376
0.0060	319	320	1	312	−7	0.0739	0.0753	0.0650	0.2142

**Table 4:** Case B: Result of simulation with the effect of mean strain omitted.

ε_a_	σ_a1_ (MPa)	σ_a2_ (MPa)	σ_m2_ (MPa)	σ_a3_ (MPa)	σ_m3_ (MPa)	D_1B_	D_2B_	D_3B_	D_B_
0.0020	246	246	0	246	0	0.0026	0.0026	0.0026	0.0078
0.0030	275	275	0	275	0	0.0109	0.0109	0.0109	0.0328
0.0040	294	294	0	294	0	0.0258	0.0258	0.0258	0.0775
0.0050	308	308	0	308	0	0.0470	0.0470	0.0470	0.1411
0.0060	319	319	0	319	0	0.0739	0.0739	0.0739	0.2218

## Data Availability

Data sharing is not applicable to this article.
